# miR-16-5p Suppression Protects Human Cardiomyocytes against Endoplasmic Reticulum and Oxidative Stress-Induced Injury

**DOI:** 10.3390/ijms23031036

**Published:** 2022-01-18

**Authors:** Rocío Toro, Alexandra Pérez-Serra, Alipio Mangas, Oscar Campuzano, Georgia Sarquella-Brugada, Maribel Quezada-Feijoo, Mónica Ramos, Martin Alcalá, Esther Carrera, Carlos García-Padilla, Diego Franco, Fernando Bonet

**Affiliations:** 1 Medicine Department, School of Medicine, University of Cádiz (UCA), 11003 Cádiz, Spain; alipio.mangas@uca.es; 2 Research Unit, Biomedical Research and Innovation Institute of Cadiz (INiBICA), Puerta del Mar University Hospital, 11009 Cadiz, Spain; 3 Cardiology Service, Hospital Josep Trueta, University of Girona, 17007 Girona, Spain; aperez@gencardio.com; 4 Cardiovascular Genetics Center, University of Girona-IDIBGI, 17190 Girona, Spain; oscar@brugada.org; 5 Internal Medicine Department, Puerta del Mar University Hospital, School of Medicine, University of Cadiz, 11009 Cadiz, Spain; 6 Centro de Investigación Biomédica en Red, Enfermedades Cardiovasculares (CIBERCV), 28029 Madrid, Spain; 7 Medical Science Department, School of Medicine, University of Girona, 17003 Girona, Spain; georgia@brugada.org; 8 Arrhythmias Unit, Hospital Sant Joan de Déu, University of Barcelona, 08950 Barcelona, Spain; 9 Cardiology Department Hospital Cruz Roja, Alfonso X University, 28003 Madrid, Spain; maribelquezada2000@gmail.com (M.Q.-F.); monica.ramos81@gmail.com (M.R.); 10 Facultad de Farmacia, Universidad CEU-San Pablo, CEU Universities, 28668 Madrid, Spain; martin.alcaladiazmor@ceu.es (M.A.); escarrera@ceu.es (E.C.); 11 Departamento de Anatomia, Embriologia y Zoologia, Facultad de Medicina, Universidad de Extremadura, 06006 Badajoz, Spain; cgp00013@red.ujaen.es; 12 Departamento de Biologia Experimental, Facultad de Ciencias Experimentales, Universidad de Jaén, 23071 Jaén, Spain; dfranco@ujaen.es; 13 Medina Foundation, Technology Park of Health Sciences, 18016 Granada, Spain

**Keywords:** miR-16-5p, ischemic dilated cardiomyopathy, reactive oxygen species, endoplasmic reticulum stress, ATF6

## Abstract

Oxidative stress, defined as the excess production of reactive oxygen species (ROS) relative to antioxidant defense, plays a significant role in the development of cardiovascular diseases. Endoplasmic reticulum (ER) stress has emerged as an important source of ROS and its modulation could be cardioprotective. Previously, we demonstrated that miR-16-5p is enriched in the plasma of ischemic dilated cardiomyopathy (ICM) patients and promotes ER stress-induced apoptosis in cardiomyocytes in vitro. Here, we hypothesize that miR-16-5p might contribute to oxidative stress through ER stress induction and that targeting miR-16-5p may exert a cardioprotective role in ER stress-mediated cardiac injury. Analysis of oxidative markers in the plasma of ICM patients demonstrates that oxidative stress is associated with ICM. Moreover, we confirm that miR-16-5p overexpression promotes oxidative stress in AC16 cardiomyoblasts. We also find that, in response to tunicamycin-induced ER stress, miR-16-5p suppression decreases apoptosis, inflammation and cardiac damage via activating the ATF6-mediated cytoprotective pathway. Finally, ATF6 is identified as a direct target gene of miR-16-5p by dual-luciferase reporter assays. Our results indicate that miR-16-5p promotes ER stress and oxidative stress in cardiac cells through regulating ATF6, suggesting that the inhibition of miR-16-5p has potential as a therapeutic approach to protect the heart against ER and oxidative stress-induced injury.

## 1. Introduction

Dilated cardiomyopathy (DCM) is one of the most common causes of heart failure (HF) and is associated with significant morbidity and mortality. From a pathophysiology perspective, DCM involves ventricular chamber enlargement and systolic dysfunction with an ejection fraction of <50% [1]. Ischemic DCM (ICM) is the most prevalent cause of DCM and leads to malignant arrhythmias and HF and is thus considered one of the most common causes of heart transplantation [2,3]. 

Decades of research have provided substantial evidence that oxidative stress plays an important role in the pathophysiology of cardiac remodeling and HF [4,5,6,7,8,9,10]. Furthermore, oxidative stress has been associated with DCM both in human and animal models [11,12,13,14]. Oxidative stress is defined as an imbalance between oxidants and antioxidants in favor of the oxidants, leading to the disruption of redox signaling and control and/or molecular damage [15]. In the heart, reactive oxygen species (ROS) play a critical role in controlling cell homeostasis in cardiomyocytes since they act as second messengers in different cellular pathways. However, ROS overproduction can become extremely dangerous to proteins, membranes, and nucleic acids, and is linked to multiple pathophysiological pathways in the heart. Protective mechanisms balancing the dangerous effects of ROS include the scavenging activity of superoxide dismutase (SOD), catalase (CAT), and glutathione peroxidase (GPx) [16,17,18].

Recent findings suggest that endoplasmic reticulum (ER) stress is intimately related to ROS production in animals [19,20,21]. Furthermore, in the last decade, ER stress has been associated with several cardiac diseases, including ischemia, DCM, and HF [22,23]. The ER coordinates the synthesis, folding, and quality control of almost all secreted and membrane proteins. Alteration in ER homeostasis causes the accumulation of unfolded and misfolded proteins in the ER lumen, leading to ER stress [24]. When ER stress occurs, the unfolded protein response (UPR) is activated to re-establish cellular proteostasis. UPR is initiated by the activation of three signaling branches, namely inositol-requiring enzyme-1α (IRE1α), protein kinase R (PKR)-like endoplasmic reticulum kinase (PERK), and activating transcription factor 6 (ATF6), which culminates in the attenuation of protein synthesis and a transcriptional response aimed at coping with the accumulation of unfolded proteins [25,26]. In normal conditions, the three transducers are kept inactive through the chaperone binding protein/78 kDa glucose-regulated protein (GRP78). In stressed conditions, GRP78 dissociates from IRE1α, PERK, and ATF6 and allows UPR activation [26]. Since protein folding is linked to ROS formation, the increment in folding load during ER stress strongly induces ROS production and exacerbates oxidative stress [27]. The activation of UPR can moderate ROS production by reducing the folding demand but also by the activation of genes encoding antioxidant enzymes [28,29]. Nevertheless, if the stress is severe or chronic, UPR assumes an adverse role and triggers apoptosis [30,31].

MicroRNAs (miRNAs) are a class of noncoding single-stranded RNA molecules with 22 nucleotides that regulate gene expression post-transcriptionally [32,33,34]. Besides their well-known role in many cardiovascular diseases and biological processes [35,36,37,38,39], miRNAs have emerged as key regulators of the ER stress response and important players in ER UPR-dependent signaling in the heart [40]. Additionally, miRNAs have been identified as potential biomarkers of DCM, cardiac remodeling, and HF [41,42]. In this context, we have previously demonstrated that miR-16-5p is upregulated in the plasma of ICM patients [43]. Our in vitro studies showed that miR-16-5p promotes ER stress-induced apoptosis, autophagy, and inflammation in human ventricular myocytes [43]. In the present study, we hypothesize that miR-16-5p might contribute to oxidative stress through ER stress induction and that targeting miR-16-5p may exert a cardioprotective role in ER stress-mediated cardiac injury. The contribution of miR-16-5p-mediated ER stress to ROS production, as well as the ability of miR-16-5p inhibition to counteract ER-stress induced cardiac injury, is tested in vitro in the human cardiomyocyte cell line AC16. The modulation of the UPR pathway in miR-16-5p-mediated ER stress is also investigated.

## 2. Results

### 2.1. Patients and Control Subjects

The baseline characteristics of the study population are presented in Table 1. There were no statistically significant differences in gender. As expected, the ICM group exhibited an increase in left atrial dimension and E/e′ ratio, compared with the control group (*p* < 0.001). Mitral annular plane systolic excursion was significantly reduced in the ICM group (*p* < 0.001) and no significant differences were observed in tricuspid annular plane systolic excursion. Both mitral and tricuspid insufficiency were only detected in ICM patients. Finally, confirming the results from our previous work [43], expression levels of miR-16-5p were increased in the plasma of ICM patients.

### 2.2. Increased Oxidative Stress in Plasma from ICM Patients

**Figure 1:**
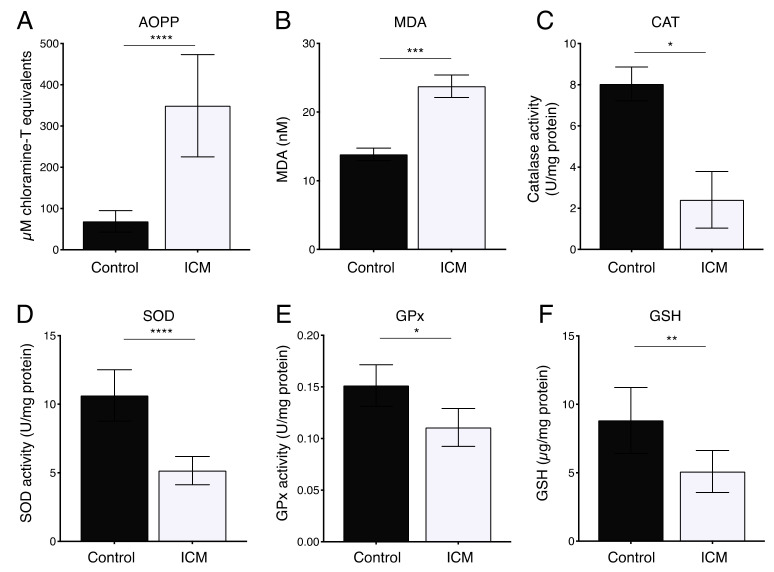
Plasma levels of oxidative stress biomarkers in patients with ICM. (**A**,**B**) MDA and AOPP levels in ICM patients (*n* = 12) are significantly higher than those in controls (*n* = 12). (**C**–**F**) Activity of the antioxidant enzymes (**C**) CAT, (**D**) SOD, (**E**) GPx, and (**F**) GSH is lower in ICM patients than in controls. * *p* < 0.05, ** *p* < 0.01, *** *p* < 0.005, **** *p* < 0.0001.

Oxidative stress has been demonstrated to be elevated in plasma from patients with ischemic heart disease and non-ischemic DCM [44,45]. We therefore assessed the oxidative stress status in ICM patients by the quantitative examination of malondialdehyde (MDA) and advanced oxidation protein products (AOPPs). As shown in Figure 1A,B, the levels of MDA and AOPP in the plasma of ICM patients were increased as compared to controls. In addition, the activity of antioxidant enzymes CAT, SOD, GPx, and reduced glutathione (GSH) were decreased in the plasma of ICM patients as compared to the control cohort (Figure 1C–F). These results confirm that oxidative stress is increased in plasma from ICM patients.

### 2.3. miR-16-5p Overexpression Induces Oxidative Stress in Human Cardiomyoblas

**Figure 2:**
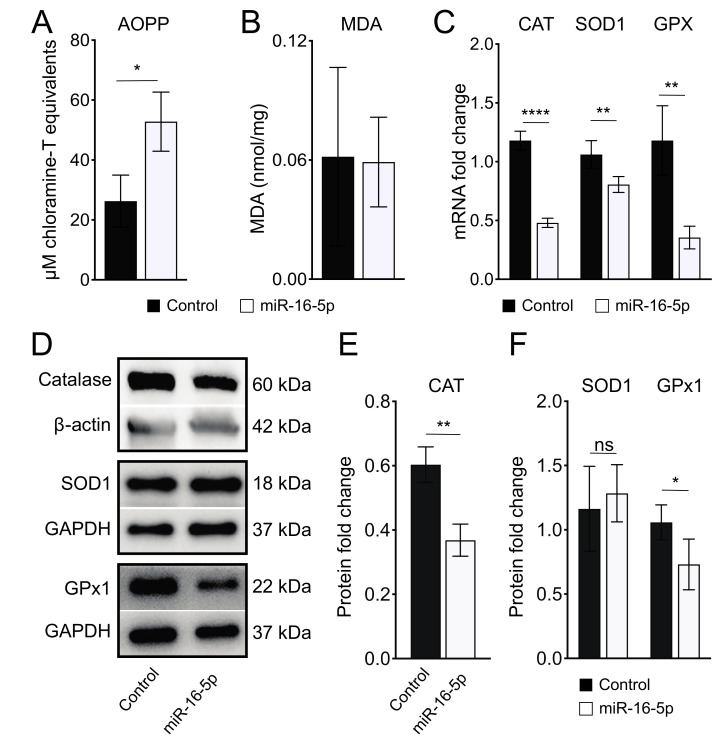
miR-16-5p overexpression causes oxidative stress in cardiomyoblasts in vitro. (**A**) AOPP determination shows increased levels in AC16 cells after miR-16-5p overexpression (*n* = 6). (**B**) The levels of MDA content are unaltered in miR-16-5p-overexpressing AC16 cells (*n* = 6). (**C**) qPCR analysis shows reduced expression levels of the antioxidant genes *CAT, SOD1,* and *GPX* in AC16 cells after miR-16-5p overexpression (*n* = 6). (**D**) Representative western blots of CAT, SOD1, and GPx1 in AC16 cells after miR-16-5p overexpression. (**E**,**F**) Densitometry analysis of western blots show reduced levels of (**E**) CAT (*n* = 5) and (**F**) GPx1 (*n* = 4). * *p* < 0.05, ** *p* < 0.01, **** *p* < 0.0001, ns: nonsignificant.

Since ER stress is closely linked to ROS generation, contributing to oxidative stress [27], we next analyzed whether miR-16-5p-induced ER stress causes oxidative stress in AC16 cells. For this purpose, we first measured AOPP and MDA levels as markers of oxidative stress in AC16 cells treated with miR-16-5p mimic. As shown in Figure 2A, AOPP levels were higher in AC16 cells after miR-16-5p overexpression than in control conditions, whereas MDA levels remained unaltered (Figure 2B). Additionally, qPCR analysis showed that miR-16-5p significantly downregulated the expression of antioxidant genes *CAT, SOD1,* and *GPX* in AC16 cells (Figure 2C). In agreement, western blot analysis also showed a significant decrease in CAT and GPx1 protein levels in AC16 cells transfected with miR-16-5p mimic over controls (Figure 2D,E). These data indicate that miR-16-5p promotes oxidative stress in human cardiomyoblasts.

### 2.4. Effect of miR-16-5p on Mitochondrial Respiration in Cultured Human Cardiomyoblasts

**Figure 3:**
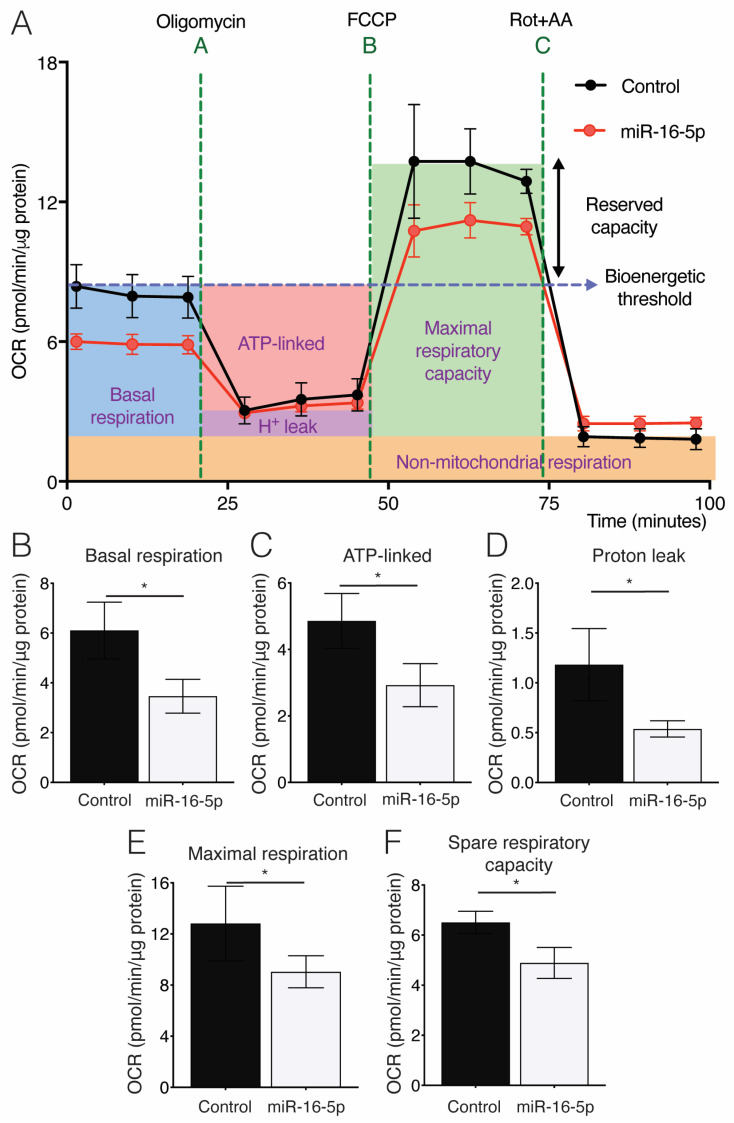
miR-16-5p overexpression leads to mitochondrial dysfunction in AC16 cells: Seahorse Cell Mito Stress Test to measure OCR in AC16 cells 24 h following miR-16-5p overexpression (*n* = 4). (**A**) OCR profile plot. (**B**) Basal respiration, (**C**) ATP-linked, (**D**) proton leak, (**E**) maximal respiration, and (**F**) spare respiratory capacity are reduced in AC16 cells after miR-16-5p overexpression. * *p* < 0.05. FCCP, Carbonyl cyanide 4-(trifluoromethoxy)phenylhydrazone; ROT, inhibitor rotenone; AA, antimycin A.

Mitochondria, especially abundant in the heart, are the major site of ROS production, and impairment of their function leads to the excessive production of ROS, contributing to cardiac pathology [46]. The acute induction of ER stress leads to mitochondrial dysfunction, enhancing ROS production in the heart [47]. Therefore, we next investigated the impact of miR-16-5p-induced ER stress on mitochondrial respiration by measuring the oxygen consumption rates of mitochondria (OCR). Overexpression of miR-16-5p induced a significant decrement in overall mitochondrial respiration as AC16 cells showed a substantial decrease in OCR after miR-16-5p transfection (Figure 3A–F). Quantification of OCR parameters determined that miR-16-5p appears to attenuate basal respiration (Figure 3B), ATP-synthesis-coupled respiration (Figure 3C), proton (H^+^) leak (Figure 3D), maximal respiratory capacity (Figure 3E), and reserve capacity (Figure 3F) in AC16 cells. These results indicate that miR-16-5p promotes mitochondrial dysfunction in human cardiomyoblasts.

### 2.5. miR-16-5p Suppression Promotes the Cytoprotective Role of ER Stress in Human Cardiomyoblasts 

**Figure 4:**
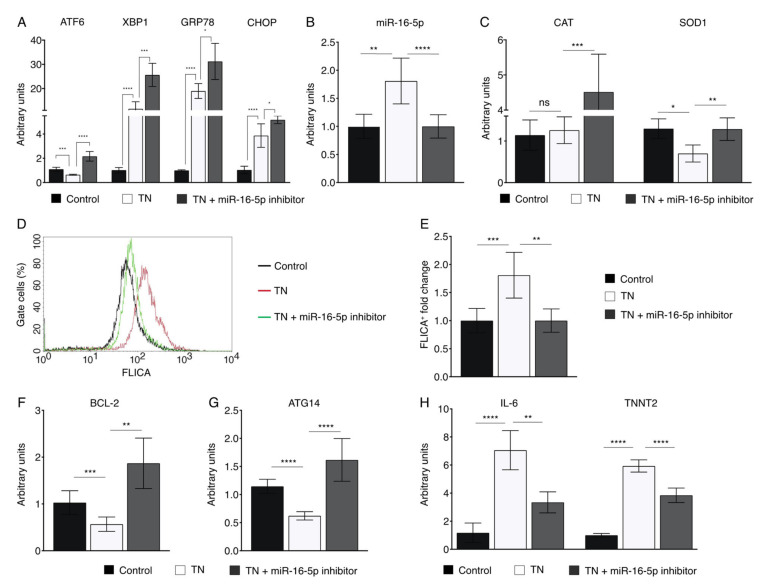
miR-16-5p inhibition reverts the damage caused by TN-induced ER stress in cardiomyoblasts. (**A**) qPCR analysis of *ATF6*, *XBP1*, *GPR78*, and *CHOP* expression in control (*n* = 5), TN-treated (*n* = 6), and TN plus miR-16-5p inhibitor-treated (*n* = 6) AC16 cells. (**B**) qPCR analysis of miR-16-5p expression in control (*n* = 5), TN-treated (*n* = 6), and TN plus miR-16-5p inhibitor-treated (*n* = 5) AC16 cells. (**C**) qPCR analysis of *CAT* and *SOD1* in control (*n* = 6), TN-treated (*n* = 6), and TN plus miR-16-5p inhibitor-treated (*n* = 6) AC16 cells. (**D**) Representative FLICA staining of AC16 cells 6 h after TN or TN plus miR-16-5p inhibitor treatment as compared to control. The numbers in the gated regions are FLICA^+^ AC16 cells (%). (**E**) Summary of D of control (*n* = 8), TN-treated (*n* = 8), and TN plus miR-16-5p inhibitor-treated (*n* = 8) AC16 cells analyzed using the FLICA Poly Caspase Assay kit. (**F**,**G**) qPCR analysis of BCL-2 and *ATF14* expression in control (*n* = 6), TN-treated (*n* = 6), and TN plus miR-16-5p inhibitor-treated (*n* = 6) AC16 cells. (**H**) qPCR analysis of *IL-6* and *TNNT2* expression in control (*n* = 6), TN-treated (*n* = 6), and TN plus miR-16-5p inhibitor-treated (*n* = 6) AC16 cells. * *p* < 0.05, ** *p* < 0.01, *** *p* < 0.005, **** *p* < 0.0001. TN, Tunicamycin.

Based on our previous work [43], we aimed to investigate whether miR-16-5p inhibition may exert a cardioprotective role against ER stress-induced injury. For this purpose, AC16 cells were treated with the ER stressor tunicamycin (TN). TN treatment provoked ER stress in AC16 cells, as demonstrated by the upregulation of *GRP78* (Figure 4A). However, the expression of markers of the IRE1α (*XBP1*) and PERK (*CHOP*) pathways were increased by TN, whereas *ATF6* was downregulated (Figure 4A). miR-16-5p inhibition reverted the TN-induced *ATF6* downregulation (Figure 4A). In contrast, upregulation of *XBP1* and *CHOP* was exacerbated by miR-16-5p deficiency (Figure 4A). Interestingly, TN also stimulated miR-16-5p expression, corroborating the data obtained in vivo (Figure 4B).

ATF6 activates the expression levels of genes encoding antioxidant proteins in cardiomyocytes [29]. Therefore, we evaluated the effect of miR-16-5p inhibition on the expression of antioxidant genes in the context of cardiac ER stress. TN treatment only significantly altered *SOD1* expression levels but not *CAT* (Figure 4C). However, when miR-16-5p was inhibited, both *CAT* and *SOD1* expression levels were significantly upregulated (Figure 4C).

Next, we evaluated the protective effect of miR-16-5p inhibition against ER stress-induced cardiomyocyte apoptosis by assessing the activation of caspase-3 and -7 using the FLICA assay. As expected, exposure to TN resulted in a significant increase in ER stress-induced apoptosis in AC16 cells. However, miR-16-5p suppression reversed the apoptosis in response to TN (Figure 4D,E). Consistently, miR-16-5p inhibition rescued impaired antiapoptotic *BCL-2* expression in AC16 cells treated with TN (Figure 4F). On the other hand, TN decreased the expression levels of *ATG14*, a key player in regulating autophagy, but this effect was rescued when miR-16-5p was inhibited (Figure 4G).

Finally, we examined the effect of miR-16-5p disruption on cardiac damage in the context of TN-induced ER stress. The inflammatory mediator *IL-6* and the cardiac injury marker cardiac troponin T (*TNNT2*) were upregulated in AC16 cells treated with TN. However, miR-16-5p inhibition significantly downregulated their expression (Figure 4H). Collectively, these results indicate that miR-16-5p suppression has a protective role against ER stress-induced cardiomyocyte damage.

### 2.6. miR-16-5p Directly Targets ATF6

**Figure 5:**
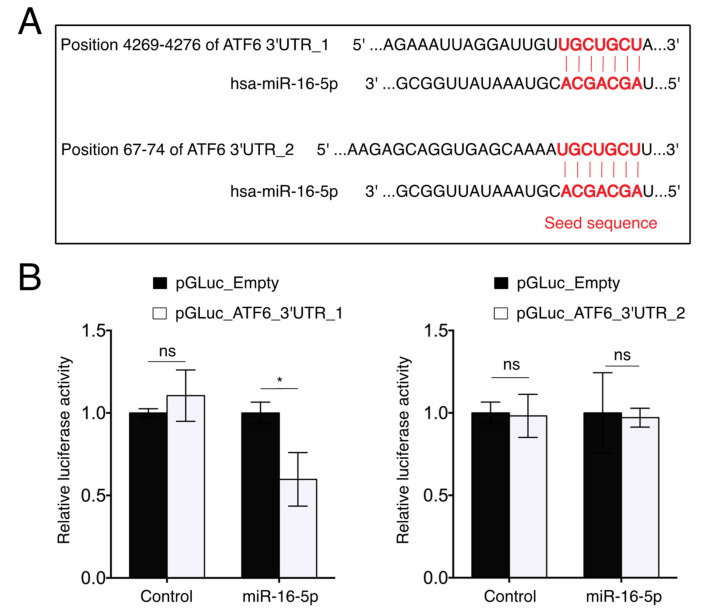
miR-16-5p targets ATF6. (**A**) Predicted miR-16-5p binding sites in the 3′UTR of ATF6. (**B**) Dual-luciferase activity assay in 3T3 cells co-transfected with the pMIR-REPORT miRNA expression reporter vector containing the ATF6 3′UTR_1 or ATF6 3′UTR_2 fragment with miR-16-5p mimic for 18 h (*n* = 3). * *p* < 0.05. ns: nonsignificant.

To explore the underlying mechanism by which miR-16-5p regulates ER stress, we investigated the target of miR-16-5p, which might modulate the UPR signaling pathway. By the prediction of new targets of miR-16-5p via TargetScan 7.2 (http://www.targetscan.org/vert_72/ accessed on 1 December 2021), we found that the 3′UTR region of human *ATF6* contained two putative miR-16-5p target sites (Figure 5A), one highly conserved (hereafter called ATF6 3′UTR_1) and another poorly conserved across different species (hereafter called ATF6 3′UTR_2) (data not shown). To verify this prediction, we performed a dual-luciferase reporter assay. The pMIR-REPORT containing the ATF6 3′UTR_1 or ATF6 3′UTR_2 was co-transfected with miR-16-5p mimics into 3T3 cells, respectively. The results showed that miR-16-5p significantly reduced the luciferase activity of the pMIR-REPORT vector containing the ATF6 3′UTR_1, whereas the luciferase activity of the pMIR-REPORT vector containing the ATF6 3′UTR_2 was unaltered (Figure 5B). These results confirmed that *ATF6* is a direct target gene of miR-16-5p.

## 3. Discussion

In the present study, we provide evidence that miR-16-5p mediates the crosstalk between ER stress and oxidative stress in ICM. In this context, we identify miR-16-5p as a crucial regulator of the ATF6-mediated cytoprotective response upon ER stress activation in AC16 cells. 

The importance of the link between ER stress and oxidative stress in the development of cardiac diseases such as ICM has been well documented [27,29]. In this regard, previous studies have reported elevated levels of the oxidative stress markers AOPP and MDA in ischemic heart diseases [44,45,48]. We first confirmed elevated oxidative stress in the plasma of ICM patients as levels of AOPP and MDA were increased, reinforcing the involvement of oxidative stress in the development of ischemic heart disease. In addition, we observed lower activity of antioxidant enzymes in the plasma of ICM patients. ATF6 is an inducer of numerous antioxidant genes in the heart during ischemia-induced ER stress in cardiomyocytes [29]. We previously reported that miR-16-5p, enriched in the plasma of ICM patients, induces ER stress, decreasing *ATF6* expression in human cardiomyoblasts [41]. Together, our observations suggest that miR-16-5p may play a role in ER stress-related ROS production in the context of ICM, impairing the cytoprotective role of ATF6. 

miR-16-5p was previously shown to be upregulated in the ischemic heart [49]. More recently, miR-16-5p was identified to be upregulated in both human and rat ventricular cardiomyocytes after ischemia/reperfusion (I/R) treatment [50,51]. Finally, miR-16 was upregulated in neonatal rat ventricular cells under oxidative stress induced by hydrogen peroxide [52]. Therefore, we next examined whether miR-16-5p may induce oxidative stress in cardiomyocytes in vitro. We showed that miR-16-5p overexpression resulted in increased levels of AOPP and downregulation of antioxidant genes’ expression in human cardiomyocytes. Thus, our data collectively indicate that miR-16-5p promotes both ER stress and oxidative stress, suggesting a link between miR-16-5p-induced ER stress and oxidative stress in cardiomyocytes. Consistently, the role of miRNAs mediating ER stress and oxidative stress during myocardial I/R injury has been already described. In 2016, Ke and colleagues showed that miR-93 protects against I/R-induced ROS generation and ER stress-mediated cardiomyocyte apoptosis [53]. 

It has been shown that ER stress induces cardiac dysfunction through the alteration of mitochondrial function in cardiomyocytes [54,55]. The fact that ER stress-mediated mitochondrial dysfunction enhances ROS production in the adult heart [46,47] prompted us to investigate whether miR-16-5p overexpression impairs mitochondrial respiration in human cardiomyoblasts. Our results showed that miR-16-5p also promoted mitochondrial dysfunction in AC16 cells, suggesting that miR-16-5p might also exacerbate oxidative stress via ER stress-induced mitochondrial dysfunction in an ICM context. 

ATF6 deficiency has been associated with mitochondria function impairment [56]. On the other hand, Jin and colleagues previously reported ATF6 as a link between ER stress and oxidative stress in myocardial ischemia [29]. This study showed that the knockdown of ATF6 in cardiac myocytes subjected to I/R increased ROS and necrotic cell death, and that *ATF6* overexpression mitigated these effects. They also observed that knocking out ATF6 in mice increased damage upon I/R, whereas AAV9-mediated *ATF6* overexpression restored ATF6 knockout heart function [29]. More recently, Blackwood and colleagues showed that the pharmacological activation of ATF6 improved ER proteostasis and decreased oxidative stress cardiac myocytes in vitro. In addition, intravenous administration of the compound 147 protected the heart from I/R damage in vivo [57]. Concordantly, we show here that *ATF6* is a direct target of miR-16-5p and that miR-16-5p inhibition reverted TN-induced *ATF6* downregulation in human cardiac cells. Therefore, our observations suggest that miR-16-5p might exacerbate oxidative stress in an ER stress-dependent manner, impairing the ATF6-mediated cytoprotective pathway in human cardiomyocytes.

**Figure 6:**
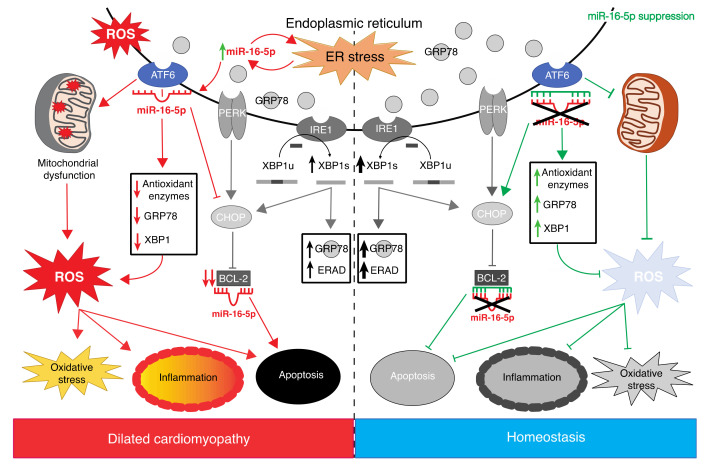
Working model for the role of miR-16-5p in the regulation of the ATF6-mediated UPR signaling in human cardiomyocytes. miR-16-5p inhibits the ER stress-induced cytoprotective response mediated by ATF6, which results in mitochondrial dysfunction and an increase in ROS production, promoting cardiomyocytes′ inflammation and apoptosis. miR-16-5p suppression activates the ATF6-mediated cytoprotective response, maintaining cell homeostasis. Arrow symbols indicate the activation pathway or signaling pathway. T symbols indicate nonactivation pathway or signaling pathway.

We next examined whether the inhibition of miR-16-5p may protect human cardiomyoblasts against acute ER stress induced by the ER stressor TN. miR-16-5p loss-of-function rescued the impaired *ATF6* expression and upregulated antioxidant genes’ expression in TN-treated AC16 cells, promoting the cytoprotective adaptation to ER stress. In contrast, the expression of *XBP1*, an indicator of IRE1α activation, was even higher when miR-16-5p was inhibited. Since ATF6 has been shown to bind to the promoter of *XBP1*, enhancing its expression [58], the upregulation of *XBP1* may be due to a combined effect of the ATF6 activation upon miR-16-5p inhibition plus the TN-induced *XBP1* expression. A similar scenario was observed in the upregulation of *GPR78*, whose expression is also regulated by ATF6. This is consistent with previous reports showing that, although the three UPR pathways are often activated together, the selective activation of some pathways together with the suppression of others can occur [59,60]. TN treatment also induced miR-16-5p expression, reinforcing our hypothesis that miR-16-5p may constitute a crucial player mediating the complex crosstalk between ATF6-mediated ER stress and ROS production in the heart. A working model summarizing the role of miR-16-5p in the ER stress and UPR response in the context of ICM is shown in Figure 6. Although TN treatment induced miR-16-5p expression, only *SOD1* expression was downregulated. The discrepancy between these results and those observed upon miR-16-5p treatment may be explained by the fact that TN promotes the expression of *XBP1*, whereas miR-16-5p impairs *XBP1* expression [43]. As XBP1 is able to regulate *CAT* downstream [61], the effect of miR-16-5p on *CAT* expression might be compensated by *XBP1* upregulation. *SOD1* expression was rescued by miR-16-5p inhibition, whereas *CAT* expression was induced, supporting the notion that miR-16-5p might exacerbate oxidative stress, regulating the expression of antioxidant genes through ATF6.

In addition, miR-16-5p suppression also protected human cardiomyoblasts against ER stress-induced apoptosis. Accordingly, the expression of the antiapoptotic *BCL-2* was reverted by miR-16-5p inhibition in TN-treated AC16 cells. Surprisingly, inhibition of miR-16-5p maximized the TN-induced *CHOP* upregulation, possibly promoted by the upregulation of *ATF6* and *XBP1* upon miR-16-5p inhibition [58]. This is in apparent disagreement with the fact that CHOP regulates ER stress-induced apoptosis by decreasing *BCL-2* expression [62]. Nevertheless, *BCL-2* is a direct target of miR-16-5p [63], indicating that BCL-2 may be also regulated in a CHOP-independent manner, as already described [64]. We finally demonstrated that indeed miR-16-5p directly targets *ATF6*, suggesting that the increased survival of miR-16-5p knockdown cardiomyoblasts upon TN treatment may be due to the combined effect of *ATF6* and *BCL*-2 gene regulatory changes. Finally, the protective role of miR-16-5p against ER stress-induced injury was confirmed as the expression levels of *IL-6* and *TNNT2*, markers of inflammation and cardiac injury, respectively, were reverted upon miR-16-5p inhibition. 

There are limitations to this study. First, the study group was relatively small; however, the included patients were strictly ICM subjects. Second, the lack of heart tissue samples is a common intrinsic feature in clinical human studies. Thus, oxidative stress markers were measured in blood samples, which are the most commonly used in clinical practice. The age of the study population showed significant differences between cohorts, which might have affected the oxidative stress status. However, Pearson correlation analysis shows no correlation between age and miR-16-5p expression in ICM patients (*r* = −0.553; *p* = 0.062), indicating that miR-16-5p expression is not age-dependent. Finally, the present study was based on in vitro experiments, and the effect of miR-16-5p suppression in vivo has not been tested. Finally, studies including a larger sample size would be needed to validate these data. 

In conclusion, we demonstrate for the first time that miR-16-5p is a key player in the ATF6-mediated crosstalk between ER stress and oxidative stress through the direct targeting of *ATF6*, and miR-16-5p inhibition protects human cardiomyoblasts against ER stress and associated damage, inducing the ATF6-mediated cytoprotective response. Therefore, miR-16-5p suppression could emerge as a promising therapeutic strategy to limit the development and progression of ICM associated with ER stress.

## 4. Materials and Methods

### 4.1. Study Population and Blood Sampling

This is a case–control study. We recruited twelve healthy controls and twelve ICM patients. Only individuals older than 18 years were included. Complete clinical information, including family and personal history, symptoms of HF, as well as pharmacological information, was acquired from each patient. Transthoracic echocardiogram and electrocardiogram were performed for all individuals. All ICM patients were verified with a coronary artery catheterization, as recommended by the European Society of Cardiology guidelines [65]. ICM was considered if significant stenosis (luminal diameter stenosis ≥50% of the left main artery or ≥75% of epicardial coronary artery) was present in one or more major coronary arteries. Plasma samples were obtained and processed as previously described [66].

This study was approved by the Ethics Committee of the Puerta del Mar University Hospital, and all patients gave their written informed consent. The ethical research principles were fulfilled following the Helsinki Declaration and the Belmont report. The study also adhered to two legal provisions governing human research and the Spanish Organic Law 15/1999 for the Regulation of Automated Processing of Personal Data.

### 4.2. Oxidative Damage Determination

We used plasma for the determination of markers of oxidative damage to lipids and proteins after the addition of 5 mM butylated hydroxytoluene as an antioxidant. To measure MDA and AOPP levels, AC16 cells were lysed with 100 μL of RIPA buffer on ice for 20 min and centrifuged for 30 min at 12,000 rpm. MDA was determined using the thiobarbituric acid reactive substances method [67]. The content of AOPP was measured according to Witko–Sarsat′s method, with minor modifications [68].

### 4.3. Enzymatic and Non-Enzymatic Antioxidant Systems

CAT-specific activity was measured by monitoring the disappearance of hydrogen peroxide at 240 nm [69]. To measure superoxide dismutase (SOD1) enzymatic activity, we adapted the method described by Bamforth [70]. The glutathione peroxidase (GPx)-specific activity assay is based on the oxidation of glutathione by GPx and measured following the disappearance of NADPH ^+^ H^+^ at 340 nm [71]. The main water-soluble, non-enzymatic antioxidant, reduced glutathione (GSH), was measured at pH 8 after the formation of a complex with the fluorescent probe o-phthaldialdehyde at an excitation wavelength of 350 nm and an emission wavelength of 420 nm.

### 4.4. Cell Culture and Transfection

AC16 cells were maintained in DMEM F12 with 10% fetal bovine serum as well as 1% penicillin/streptomycin, at 37 °C and 5% CO_2_. The overexpression of miR-16-5p was performed as previously described [41]. Inhibition of miR-16-5p was performed by transfection of 180 pmol of mirVana^®^ has-miR-16-5p specific inhibitor (Thermo Fisher Scientific, Carlsbad, CA, USA) using Lipofectamine^®^ RNAiMA Reagent (Invitrogen, Carlsbad, CA, USA), following the manufacturer′s recommendations. Cells were treated with 2 µM of tunicamycin (TN) (Sigma-Aldrich, St. Louis, MO, USA) for 6 and 18 h to induce the ER stress.

### 4.5. RNA Isolation and Real-Time Quantitative RT-qPCR

The MagMAX™ *mir*Vana Total RNA Isolation Kit (Thermo Fisher Scientific, Vilnius, Lithuania) was used to isolate the total RNA from AC16 cells. RNA was reverse-transcribed using a High-Capacity cDNA Reverse Transcription Kit (Thermo Fisher Scientific, Waltham, MA, USA), following the manufacturer’s instructions. For miR-16-5p, U6 snRNA, and GAPDH gene expression analysis, a commercial Taqman Gene Expression Assay (Applied Biosystems, Pleasanton, CA, USA) was used. Relative quantification of mRNA levels (primer pairs shown in Table 2) was executed using 6.5 ng of cDNA, forward and reverse primers at 100 nM each, and PowerUp SYBR Green Master Mix (Applied Biosystems, Foster City, CA, USA). The mRNA levels were normalized to GAPDH. The expression levels were calculated by the Livak formula, and the expression of the control group was set to 1.

### 4.6. Western Blot Analysis

Transfected AC16 cells were harvested in RIPA, and protein concentration was determined using a Pierce BCA protein assay kit (Thermo Fisher Scientific, Rockford, IL, USA). Equal amounts (20 µg) of protein were loaded, electrophoresed on 8–12% SDS-PAGE. Membranes were incubated with specific monoclonal anti-Catalase (diluted 1:1000; Cell Signaling, Beverly, MA, USA), anti-SOD1 (diluted 1:1000; Cell Signalling, Beverly, MA, USA), anti-GPx1 (diluted 1:1000; Cell Signalling, Beverly, MA, USA), anti-GAPDH (diluted 1:5000; Invitrogen, Rockford, IL, USA) and monoclonal anti-β-Actin−Peroxidase antibody (diluted 1:50,000; Sigma-Aldrich, St. Louis, MO, USA) overnight at 4 °C with constant agitation. Following incubation, membranes were washed and incubated for 1 h at room temperature with HRP-linked secondary anti-mouse IgG (diluted 1:1000; Cell Signaling, Beverly, MA, USA) or anti-rabbit IgG (diluted 1:10,000; Cell Signaling, Beverly, MA, USA). After washing, immunoreactive bands were visualized using Clarity Western ECL Substrate (Bio-Rad, Hercules, CA, USA). The immunoreactive bands were analyzed using a lab analysis software imaging densitometer (Bio-Rad, Hercules, CA, USA). The density of each band was evaluated with Image Lab analysis software (Bio-Rad, Hercules, CA, USA). β-Actin and GAPDH were used as loading controls and values were normalized to the signals obtained with control samples.

### 4.7. Oxygen Consumption Rate (OCR) Measurement

AC16 cells were seeded in each well of a Seahorse XF-24 plate and transfected as described above. The Seahorse XFe24 Extracellular Flux Analyzer (Agilent Technologies, Santa Clara, CA, USA) was used to measure OCR in AC16 cells. Cells were transfected in customized Seahorse 24-well plates and infected as described above. Then, 24 h post-transfection, the cells were incubated for 1 h in XF Assay Medium (Seahorse Bioscience, Santa Clara, CA, USA) plus 5 mM glucose for a typical bioenergetic profile; the OCR of basal respiration was initially measured, followed by exposure to 1 µM oligomycin (an ATP synthase inhibitor), which allowed the detection of the amount of O_2_ consumed by ATP synthesis, H^+^ leak, and other oxidases. Then, 1 µM of the uncoupler carbonyl cyanide 4-(trifluoromethoxy)phenylhydrazone (FCCP) was added to quantify the maximal respiratory capacity, followed by 1 µM of rotenone and 1 µM of antimycin A, a mix of inhibitors of the complexes I and III of the mitochondrial electron transport chain (ETC), which fully depleted mitochondrial O_2_ consumption. OCR data were calculated using Wave software v. 2.6.1 (Agilent Technologies, Santa Clara, CA, USA), and data were normalized per microgram of protein [72].

### 4.8. Luciferase Reporter Assay

ATF6 3′UTR constructs were PCR-amplified and cloned into the pMIR-REPORT vector. Then, 3T3 fibroblasts (ATCC) were co-transfected with 100 ng of the ATF6 luciferase vector and 300 ng of pcLux vector control for internal normalization. Luciferase activity was measured 18 h after transfection by using the Pierce Gaussia Luciferase Flash Assay Kit (Thermo Fisher Scientific, Rockford, IL, USA) and normalized to pcLux vector control by using the Pierce Cypridina Luciferase Flash Assay Kit (Thermo Fisher Scientific, Rockford, IL, USA). In all cases, transfections were carried out in triplicate.

### 4.9. Statistical Analysis

Data are expressed as mean ± SEM and *n* denotes the number of replicates for each experiment. Outliers were identified through the Rout method, using a Q = 1%. The normal distribution of each variable was verified with the Shapiro–Wilk test. Statistical differences (*p* < 0.05) between the experimental groups were assessed using a two-tailed, unpaired Student′s *t* test for Gaussian distributions. For non-Gaussian distributions, a Mann–Whitney non-parametric test was used. All the statistical analyses were performed using GraphPad Prism 9.0 software (San Diego, CA, USA).

## Figures and Tables

**Table 1:** Baseline characteristics of patients with ICM and controls.

Variable	Healthy Control (*n* = 12)	Ischemic DCM (*n* = 12)	*p*-Value
Age (years) ^a^, means ± SD	45.2 ± 14.4	63.0 ± 8.8	0.006
Sex (male)	62.5%	66.7%	ns
BMI (Kg/m^2^) ^a^, means ± SD	22.9 ± 3.51	29.0 ± 2.9	0.003
LVEF (%) ^a^, means ± SD	68.5 ± 6.05	34.0 ± 5.8	<0.001
LVEDD (mm) ^b^, means ± SD	45.7 ± 5.3	61.7 ± 6.5	<0.001
LVESD (mm) ^a^, means ± SD	28.0 ± 4.2	46.2 ± 15.5	0.004
LA (mm) ^a^, means ± SD	36.6 ± 6.9	47.2 ± 5.9	0.002
Sphericity index ^a^, means ± SD	0.6 ± 0,03	0.7 ± 0.04	0.03
TAPSE (mm) ^a^, means ± SD	19.09 ± 4.37	21.44 ± 4.21	ns
MAPSE (mm) ^a^, means ± SD	18.77 ± 2.88	10.98 ± 2.24	<0.001
MI	-	10 (83.4%)	0.001
TI	-	6 (50%)	0.02
E/e′ ratio ^b^, means ± SD	7.59 ± 1.59	18.12 ± 7.62	<0.001
NYHA class	I	II	<0.001
miR-16-5p expression levels (log_2_) ^a^	7.522 ± 0.43	8.232 ± 0.52	0.005

BMI, body mass index; LVEF, left ventricular ejection fraction; LVEDD, left ventricular end-diastolic diameter; LVESD, left ventricular end-systolic diameter; LA, left atrial dimension; MI, mitral insufficiency; TI, tricuspid insufficiency; E/e′, ratio of mitral early diastolic flow velocity over tissue Doppler lateral mitral annular lengthening velocity; NYHA, New York Heart Association; ns: nonsignificant. ^a^ Student’s *t* test; ^b^ Mann–Whitney U test.

**Table 2:** Quantitative real-time polymerase chain reaction primer pair sequence.

Gene	Forward	Reverse
*ATF6*	5′-AATACTGAACTATGGACCTATGAGCA-3′	5′-TTGCAGGGCTCACACTAGG-3′
*ATG14*	5′-TGGGGACTACTCTGCCTACTACA-3′	5′-GGGTTACTCTGCTCCATGTCA-3′
*BCL-2*	5′-AGCACGTGCACAGCTTCA-3′	5′-GTCCACGGGTGAAACAGC-3′
*CATALASE*	5′-TCTGGACAAGTACAATGCTGAGA-3′	5′-TAAGCTTCGCTGCACAGGT-3′
*CHOP*	5′-TCACCACACCTGAAAGCAGA-3′	5′-TCTTGCAGGTCCTCATACCA-3′
*GAPDH*	5′-AGCCACATCGCTCAGACAC-3′	5′-AATACGACCAAATCCGTTGACT-3′
*GRP78*	5′-AATGACCAGAATCGCCTGAC-3′	5′-ATGCGCTCCTTGAGCTTTT-3′
*GSH*	5′-CCTGCTAGTGGATGCTGTCA-3′	5′-TCATCCTGTTTGATGGTGCT-3′
*IL-6*	5′-GATGAGTACAAAAGTCCTGATCCA-3′	5′-CTGCAGCCACTGGTTCTGT-3′
*SOD1*	5′-TCCATGTTCATGAGTTTGGAGAT-3′	5′-CCCACCGTGTTTTCTGGATA-3′
*TNNT2*	5′-GGCTGCAGTGGCTACAGG-3′	5′-CTGTCACCAGGCAATACAGC-3′
*XBP1*	5′-TGCGTAGTCTGGAGCTATGGT-3′	5′-CCCGACAGAAGCAGAACTTT-3′

## Data Availability

In-data transparency is guaranteed. The datasets generated during and/or analyzed during the current study are available from the corresponding author on reasonable request.
